# Intelligence

**Published:** 1827-07

**Authors:** 


### INTELLIGENCE.

### MONTHLY REPORT OF PREVALENT DISEASES.

There has been prevalent during the present season a greater disposition than usual to an intermittent or remittent form of fever: agues, indeed, of well-marked character have not been unfrequent in the metropolis, and some parts of the country adjacent. But, independent of these circumstances, common *continued* fever, as it is called, has in many instances assumed a remittent character after the second week. This change, however, has not appeared to us to be allied to ague so much as to hectic, depending generally, if not invariably, upon local disease, which, having taken place in the course of the fever, now *sets up for itself.* The most frequent seat of such local mischief is the abdomen, especially the ileum and the portion of the colon next to it, in which ulcerations are known to be so common. In some instances we have seen the supervention of these disorganisations first manifested by violent rigors, a symptom which has always denoted a very formidable disease.

---

### COLLEGE OF PHYSICIANS.

*Question whether Fellows of the College of Physicians ought or ought not to meet Physicians who are not Licentiates.*

Sir,—I request the favour of your inserting the accompanying letters, and subjoined observations, in the next Number of the London Medical and Physical Journal.

I am, Sir, your obedient humble servant,

*To Dr. Macleod.* EDWARD HARRISON.

*Copy of* Dr. Harrison's *Letter to* Dr. Chambers.

7, *Holles-street, Cavendish-square; May* 12*th*, 1827.

Sir,—I was not a little surprised, on my return to Quebeck-street last Sunday evening, to learn that you had formally refused to meet me in consultation, because I had not received a licence to practise medicine from the London College of Physicians.

As the delicate sufferer was, at the time, in the greatest possible danger, I leave you to form your own conclusions upon the humanity and propriety of declining to give assistance to an afflicted fellow creature, in compliance with a capricious and untenable by-law.

To the patient and to myself the determination was fortunate, because it had led the parents, before my arrival, to procure the assistance of an experienced and able physician. This gentleman has, like myself, thought proper not to apply for the College licence, and yet he assures me that the members of your corporation do not object to consult with him, whenever their services are wanted: so true is it, that it may suit them at times to enforce the rigid observance of a by-law, and at other times to leave it entirely to individual discretion.

To enter into a minute investigation of the supposed grounds of your refusal, would lead me far beyond the limits of an ordinary letter. It will be sufficient for my present purpose to state, that neither the late Dr. Baillie, nor your colleagues Drs. Warren, Turner, or Paris, ever ventured upon such a measure, when their medical services were requested along with mine; and it would, perhaps, have been more suitable to a person in your professional station to have imitated their example, than to have formed a rule for yourself.

As far as concerns me individually, it is really a matter of perfect indifference whether I am in future to meet in consultation with the fellows of your College, or am to lose their services in cases of danger or obscurity. London happily contains many physicians out of the pale of your corporation, in whose skill invalids may safely confide.

Under this impression, my first determination was wholly to overlook the contents of your note, addressed to the mother of my patient; but, on referring to the purport of it, a few nights since, in a large party of physicians, who, in the phraseology of your College, are denominated "alieni homines," I became convinced of my error. Indeed, it now appears to me that, in following the bent of my inclination, I should have neglected the duty I owe to my alma mater the University of Edinburgh, and to my brethren the "alien" physicians established throughout the British dominions, as well as to the public at large.

Deeply interested in the questions at issue between the medical graduates of England and of all other countries, I shall now call your attention to some of the reasons which have led me uniformly to resist the arrogated powers of the London College. In opposing them, I am neither influenced by hostility nor prejudice. My chief aim is to relieve myself and brethren from the degradations imposed upon us.

Among the reasons which have influenced me to adopt my present course, it will be sufficient to state—first, that, unless I have been misinformed, every candidate for your licence is obliged, on his bended knees, to swear obedience to the laws and regulations of the College, though, by a refinement in legislation, as far as I know peculiar to yourselves, he is not suffered to read them either before or after he has complied with the oath. If such be the case, I cannot help giving it as my deliberate opinion that the ceremony is equally dishonourable to the parties who require, and to those who submit to this preposterous exhibition.

Secondly. Another insuperable objection to the College licence is founded on your arbitrary and illegal by-laws. According to my interpretation of the medical statutes, the College of Physicians is equally open to the graduates of every university. It possesses no distinction of rank, though the highest has, by a series of encroachments, been limited to the physicians of Oxford and Cambridge, while a lower grade has been forced upon all other physicians.

These are some of the numerous objections which I feel, and which make it impossible for me, under the present constitution of the College, to apply for their licence. Should the College still be of opinion, as they formerly professed to maintain, that they can legally compel the acceptance of a licence, or the discontinuance of practice, I beg them to be assured that I am perfectly ready to try the question, whenever they may think proper to afford me the opportunity. I must, however, in the mean time, strongly remonstrate against the custom of endeavouring to obtain their object by a course injurious to medical science, and prejudicial to the community.

You may possibly be aware that I formerly stated the same sentiments to Dr. Baillie, and after his death to Dr. Turner. I did not omit on either occasion to add, that the fellows were, in my opinion, highly culpable in making regulations which they dare not attempt to enforce in a court of law.

As my sentiments remain unaltered, I embrace the opportunity which you have afforded me, to renew my offer through you to the College. Should the challenge be at length accepted, I pledge myself to carry the suit to a full hearing and final decision.

In repeating my offer for the third time, I desire to remind you that I have hitherto been content to assert my own privileges and independence, when they were unnecessarily assailed. But, after so many provocations, I now think myself called upon openly to claim, for myself and colleagues, all the rights and privileges of British subjects, agreeably to the union of the two kingdoms. To an Englishman, it appears to be more than absurd and ridiculous that he should be supposed to have lost any of his natural rights by visiting another portion of the same kingdom merely to qualify himself for the duties of a profession, the knowledge of which he could no where acquire in his own part of the country.

If the fellows shall still think fit to decline the contest, an enlightened public cannot fail to appreciate their real motives, however they may be disguised or concealed.

As for the graduates of my order, they will not be slow to perceive the folly of connecting themselves with an incorporation, from which they must afterwards expect to receive only marks of neglect, of opposition, or of humiliation.

I think myself entitled explicitly to inquire from you, on this occasion, whether, in refusing to meet me in consultation, you considered yourself as acting discretionally, or under an indispensable obligation imposed on you by the by-laws of the College.

I beg leave to add, in conclusion, that, unless I receive a satisfactory answer in the space of a month, either from you or the College, to the several allegations contained in this letter, I shall feel it my duty to publish it, for the information and guidance of my brethren, wherever they may be situated.

I am, Sir, your obedient humble servant,

(Signed) EDWARD HARRISON.

*To Dr. Chambers, Brook-street.*

*Copy of* Dr. Chambers' *Answer to* Dr. Harrison's *Letter.*

*Brook-street; May* 14*th*, 1827.

Sir,—I have to acknowledge the receipt of your letter dated the 12th of May, which only reached me this afternoon.

In answer to it, I beg leave to state that I do not feel myself called upon to enter into the discussion of the questions which you conceive to be at issue between the College of Physicians and yourself.

I have only to say as to myself, that, in refusing to meet you in consultation, I acted in obedience to a positive regulation of the College; and that it is a matter of indifference to me whether you publish your letter on the subject or not.

I am, Sir, your most obedient, humble servant,

(Signed) W. F. CHAMBERS.

*To Dr. Harrison.*

---

Although Dr. Chambers declares, that, "in refusing to meet me in consultation, he acted in obedience to a positive regulation of the College," will he venture to maintain that he has never evaded this *positive regulation*, or by-law, during his connexion with that body? I have, as already observed, been joined in practice with no fewer than four fellows during my short residence in London. I may now add, that from Dr. Chambers alone have I encountered a refusal. I have also said, that the alien physician, an old metropolitan practitioner, who supplied the Doctor's place, is in the constant habit of meeting the fellows professionally. After stating these facts, I shall not expiate further upon the glaring incongruities and absurdities of the fellows, but leave them to explain their motives, and to form their own justification.

2dly. Do the fellows ever decline to consult with surgeons, on cases strictly medical? Physicians had formerly the whole management of constitutional diseases entrusted to them; and were also applied to, as the dernier resort, in surgery. But so completely are the tables now turned in these respects, that, while the surgeon openly beards the doctor in medical practice, he is jealous of the smallest encroachment upon his own department. Many examples of recent date might be given in support of these assertions. As regards the former, the reader cannot have forgotten that two individuals, of the highest medical and surgical rank, were lately in conjoint attendance, for several successive weeks, upon two distinguished and very exalted characters. One of the cases was purely medical, and the surgical treatment of the other was so inconsiderable, that the surgeon could only be wanted for his medical skill.

3dly. According to present usage in this country, the ordinary practice of physic is almost entirely confided, in the first instance, to the family apothecary. The physician is only thought of when the case becomes alarming or tedious. After his introduction, their visits are continued in accordance, and the two share the responsibility between them.

4thly. Upon what justifiable grounds, then, can the fellows refuse to be united in consultation with the "independent physician," whilst they have no hesitation in freely consulting with the surgeon and apothecary.

5thly. In a colloquial conversation with the late Dr. Baillie, so long ago as the month of June, 1821, while we were engaged upon the case of a young lady, I fully explained my opinion of the London College of Physicians, as alluded to in my letter to Dr. Chambers. This was the third patient, after my arrival in London, who had called for our joint assistance. As the Doctor had never omitted, on former occasions, to recommend my application for the College licence, I determined at this interview, if a good opening occurred, to assign my reasons for declining to comply with his urgent solicitations. The opportunity being given, I avowed it, as my deliberate conviction, arising from legal inquiries and a careful investigation of the subject—

1st. That the College of Physicians is, according to the laws of the realm, and charter of King Henry the Eighth, equally open to the medical graduates of every university. I added, that it was, in point of fact, conducted upon this principle, from the first establishment in 1523 to about the middle of the last century, including a period of more than 200 years.

2dly. That, at this eventful era, a predominant party of Oxford and Cambridge physicians, unfortunately for medical science and the true interests of their professional, had the temerity to narrow the by-laws, in order to promote their own selfish views. On referring to the exact time when these regulations were enacted, we are led to believe that they were chiefly intended to check the rising prosperity of the University of Edinburgh. Had the College formed their excluding by-laws anterior to the British union, something might perhaps have been advanced in extenuation of their conduct; though, in as much as the healing art is the production of no particular soil, it would be absurd to attempt to confine its cultivation within the limits of any district. But no sooner were the two nations consolidated into one kingdom, than it became the bounden duty of every citizen to efface local distinctions, and promote harmony through the land.

3dly. That the College was extremely culpable in making by-laws, which they durst not endeavour legally to enforce.

4thly. That it was due to themselves, and to the physicians of my order, either to try the validity of their present regulations, or to make such as they would be able to defend.

5thly. That, fully satisfied with the stability of my own position, I was ready, whenever the College were pleased to attack my station, to defend it with legal and constitutional weapons.

Such was the purport of my conference with Dr. Baillie, at our last interview; and a similar, though less extended, conversation took place, in the year 1824, between Dr. Turner and myself. Having subsequently been met in consultation both by Drs. Paris and Warren, judge of my surprise on receiving a positive refusal in the person of Dr. Chambers.

Having given insertion to the above correspondence, in compliance with the request of Dr. HARRISON, we take leave to offer a few observations upon it. Two questions naturally suggest themselves from its perusal: first, whether the Fellows of the College of Physicians ought, or ought not, to meet in consultation physicians who are not Licentiates? and, secondly, whether the College ought, or ought not, to proceed against those who refuse to submit to their jurisdiction?

It appears to have been clearly established by repeated decisions in a court of law, that the College of Physicians have power to regulate the practice of medicine in London and its environs, and to levy certain fines upon those who practise without a license. This has no reference whatever to the university at which the degree has been obtained, but is a general privilege of calling before them every individual who may practise as a physician in London. The circumstance of the university at which he has been educated determines whether the individual is to be admitted as a fellow or licentiate; but a degree merely, although from either of the English universities, gives no power to practise in the metropolis. Most physicians, when they come to settle in London, so far acquiesce in this view as to present themselves for examination, and look upon their doing so as a preliminary step towards entering into business. The College, in exercising this privilege, stand pledged in this respect to those who submit to their authority, that they are bound to extend to them, as far as possible, the advantages which they profess to have bestowed. It is, therefore, obviously both the duty and the interest of the College to make a distinction between those physicians who have a license and those who have not. It is probably with this view that the regulation was established, prohibiting fellows from meeting those who are not licentiates. Dr. Harrison gives the names of several fellows who, notwithstanding this regulation, have met him in consultation. Now, we cannot but think that the College do an act of great injustice, both to those fellows who act up to their regulations, and still more to the licentiates, whose interests are thus directly injured, in allowing their own members (whom surely they can control) to recognise equally those who acknowledge and those who contemn their authority.

With regard to the second question, its answer is involved in that of the first. If they are instituted the guardians of medical practice, and, acting upon this, subject to examination, and to the payment of certain fees, those who wish to practise *legally*, they ought undoubtedly to proceed against those who prefer practising in a manner which the College holds to be *illegal*.—Nay, more, they *must do so*: otherwise, physicians will no longer acknowledge a power,—where the acknowledgment affords no protection, and the refusal to do so incurs no penalty.

Why the College do not at once vindicate their authority, by prosecuting those who practise without a license, is a question which it is not for us to answer. We have been told that the requisite proof of practising is not always to be obtained, and also that the penalty incurred by the offender is inadequate to the prevention of the offence. But, in the present instance, the former difficulty can scarcely exist, as Dr. Harrison, if sincere in his wish to bring the matter to a legal decision, will admit the fact of his practising;* and, with respect to the latter, we must be allowed to say that it will be time enough to insist upon the inadequacy of the fine when it has been actually levied.

It is notorious that there is more than one physician now practising in despite of the College; and it is imperative on them, therefore, to exercise whatever power they have, or else the example will be followed by others, and the number who take their license will very speedily diminish.

*June 20th.*

* We believe that, to prove the fact of any one practising as a physician, it is generally necessary to bring forward one or more of his prescriptions.—In the present case, where Dr. Harrison thus publicly acknowledges that he does practice, we cannot suppose any other evidence to be requisite.

### COLLEGE OF SURGEONS.

The petition respecting the College of Surgeons has at length been presented to the House of Commons, and, as the subject must be interesting to most of our readers, we subjoin the particulars from the *Times* of Thursday, June 21.

Mr. WARBURTON rose to move for the production of certain papers connected with this establishment. In the year 1797, application was made by this corporation for an Act, which, among others, contained the extraordinary clause of securing to themselves the monopoly of lecturing on surgery. This clause was however, as it ought to have been, scouted. Ever since that time, however, they have been endeavouring, by by-laws, to get this power secured to them, which had appeared so monstrous to Parliament; and the persons composing the council, being for the most part connected with hospitals, found it their interest to support these by-laws. They had passed a law by which they refused to acknowledge any other schools of surgery than those of London, Dublin, Edinburgh, Glasgow, and Aberdeen. If a person attended a provincial hospital, he must produce a certificate of two years: if it should be a London hospital, one year was sufficient. Now two out of these five schools notoriously afforded no facilities for the study of anatomy, in consequence of the difficulty of obtaining subjects for dissection. There remained, therefore, only three schools for the study of anatomy. The College of Surgeons also refused to take any certificate of attendance at foreign schools, however distinguished they might be. Another ground of complaint was, that the Hunterian museum and library, which had been purchased for the College with the public money, were open to the public only for two days in the week. Then, again, there was no catalogue of the library, without which the library itself was nearly useless. He likewise understood that some manuscripts left by Mr. Hunter had been voluntarily destroyed by fire; a proceeding which required some explanation. The honourable member concluded by moving for a return of all sums of money granted by government to the College, from 1799 to the present period, for the purchase of the Hunterian Museum, and for the repairs of the College; also for a return of the number of persons rejected upon examination; and likewise for a return of the receipts from fees and other sources, and expenditure of the College.

Mr. W. SMITH said, he was aware that another honourable member had been requested to present the petition which the honourable member had that evening brought under the notice of the House; but he declined to do so, because he found, upon inquiry, that almost every charge preferred by the petitioners rested upon no better foundation than feelings of personal pique. He was convinced that, whenever the investigation which was called for should take place, the College would satisfactorily rebut all the accusations which had been brought against them. As to what had fallen from the honourable member with respect to a catalogue, he could state from his own knowledge, that Mr. Cliff, the distinguished anatomist, was preparing one.

Mr. PEEL said, he was a trustee of the Hunterian Museum, and in justice to the council of the College he was bound to state, that having, before he left office, discussed with them every complaint preferred in the petition, they professed their willingness to remove every one of them that appeared to be reasonable. He went over the complaints with the council *seriatim*, and for some of those now preferred there no longer existed any foundation; for the grievances (real or supposed) had at once been redressed. One of the complaints regarded the entrance to the college by a particular door; one party asserting their right to enter by it, and another party disputing that right. He advised the council to concede the point, and his advice was followed. With respect to the funds, he stated his opinion that they ought, like other great public bodies, to publish a quarterly account of the moneys received by them, and of the manner in which they had been appropriated: the council at once agreed to what he proposed. A catalogue was being prepared by one of the most able men who could be selected for such a task, and the work was already in a state of great forwardness. It was owing to the preparation of this catalogue that strangers were not admitted at present to the library oftener than twice a-week, but the moment it was completed they would be admitted four times a-week. The examinations which took place at the College were not matters of such light importance as the honourable member considered them. They were, in fact, very severe scrutinies, and the instances of rejection were numerous. The examinations, too, were conducted openly, and not in private, as the honourable member had asserted. The destruction of Mr. Hunter's manuscripts was the act of that gentleman's executor, who thought it essential to his fame that they should be destroyed. The council wished to preserve them, but found that they had no legal power to interfere. The right honourable gentleman then read an extract from a memorial which had been drawn up in favour of the College by Mr. Brodie, Mr. Travers, Mr. Davies, and Mr. Green, which spoke in the highest terms of approval of the manner in which the institution was conducted. There was one point of great importance connected with this subject: he alluded to the difficulty of procuring subjects for anatomy, without which it was impossible the science could be taught. It was in vain to attempt to supply the want of human subjects by figures formed of wax or wood. The difficulty of procuring subjects resulted from feelings which it was impossible not to respect, particularly among the poorer classes. The subject was one which had occupied much of his attention, because he was aware that it constituted an obstacle to the advancement of sciences of the utmost importance to the human race. A person destined for the surgical profession, instead of receiving his education in London or Edinburgh, was now compelled to resort to Paris for that purpose. The law, as it at present stood, by enacting that the bodies of murderers only should become subjects for dissection, encouraged the existing prejudices of the people. This was the opinion of an eminent surgeon, Mr. Hodson, who practised in the country, but was inferior to none of his competitors in talent. It had occurred to him that it would be an improvement of the present system to introduce an Act of Parliament, to provide that the bodies of all persons convicted of felonies, who should die in prisons or other public establishments, should be delivered up for dissection. If this were considered an aggravation of punishment, it might, in the acknowledged difficulty of finding a substitution for the punishment of death, be employed as a means of mitigating the severity of the criminal code. The right honourable gentleman concluded with expressing his intention to accede to the motion, not because there was the slightest ground of accusation against the College, but because he thought Parliament had a right to be informed of the proceedings of an institution which had received a grant of public money.

Sir J. Yorke suggested the propriety of passing an Act of Parliament to allow people to sell their own bodies, and those of their relatives.

After a few words from Lord Binning, Mr. H. Gurney, and Mr. S. Bourne, the motion was agreed to.

---

### NEW STYPTIC.

At a meeting of the members of the Medico-Botanical Society, holden lately, (Sir James M'Grigor, the president, in the chair,) Mr. Frost brought before their notice a specimen of a plant termed by the inhabitants of Peru *Matica*, and used by them as an external styptic. It appears to be a species of Piper. Its leaves are of a cordate shape, and very tomentose on their under surfaces. They are coarsely pulverised, and in this way externally applied. We hope, at some future period, to lay before our readers some further account of it.

---

Dr. Babington has been appointed a Fellow of the Royal College of Physicians. In this nomination, the College and the new Fellow mutually give and receive honour.

### METEOROLOGICAL JOURNAL,

*From May 20th, to June 20th, 1827.*

By Messrs. HARRIS and Co. Mathematical Instrument Makers, 50, High Holborn.

| May | Rain gauge. | Moon. | Thermom. 9 A.M. | Thermom. MAX. | Thermom. MIN. | Barometer. 9 A.M. | Barometer. 10P.M. | De Luc's Hygrom. 9 A.M. | De Luc's Hygrom. 10P.M. | Winds. 9 A.M. | Winds. 10P.M. | Atmospheric Variations. 9 a.m. | Atmospheric Variations. 2 p.m. | Atmospheric Variations. 10 p.m. |
|---|---|---|---|---|---|---|---|---|---|---|---|---|---|---|
| 20 | | | 63 | 70 | 53 | 29.93 | 29.94 | 77 | 76 | E | W | Fair | Fine | Fine |
| 21 | | | 60 | 72 | 55 | 30.00 | 30.03 | 73 | 74 | NE | W | — | — | — |
| 22 | | | 63 | 64 | 55 | 29.99 | 29.94 | 80 | 89 | WSW | W | Cloudy | Rain | Cloudy |
| 23 | .20 | | 64 | 69 | 52 | 29.88 | 29.59 | 83 | 92 | WSW | SW | — | — | Rain |
| 24 | | | 57 | 60 | 45 | 29.31 | 29.27 | 87 | 88 | W | WSW | Cloudy | Cloudy | — |
| 25 | .55 | ● | 52 | 60 | 48 | 29.24 | 29.28 | 90 | 87 | W | SW | Rain | Rain | Cloudy |
| 26 | | | 53 | 64 | 49 | 29.33 | 29.41 | 90 | 87 | SW | SW | — | Cloudy | — |
| 27 | .10 | | 59 | 66 | 54 | 29.46 | 29.54 | 82 | 88 | WSW | SSW | Fair | Rain | — |
| 28 | | | 60 | 67 | 56 | 29.58 | 29.69 | 88 | 92 | SW | SW | Cloudy | Cloudy | — |
| 29 | | | 60 | 66 | 52 | 29.66 | 29.67 | 85 | 90 | SW | SW | — | — | — |
| 30 | | | 63 | 70 | 57 | 29.75 | 29.69 | 84 | 78 | SW | SW | — | — | Fine |
| 31 | | | 63 | 70 | 52 | 29.57 | 29.67 | 78 | 83 | SSW | SW | — | Fair | — |
| June | | | | | | | | | | | | | | |
| 1 | | | 55 | 66 | 52 | 29.57 | 29.68 | 88 | 73 | SW | W | Sl. Rain | — | Cloudy |
| 2 | .12 | ☽ | 56 | 65 | 45 | 29.54 | 29.55 | 92 | 85 | SSW | W | Rain | Rain | Fine |
| 3 | | | 58 | 64 | 51 | 29.67 | 29.76 | 82 | 76 | W | WSW | Fair | Show'ry | Cloudy |
| 4 | | | 56 | 66 | 54 | 29.78 | 29.85 | 76 | 78 | W | WNW | — | — | — |
| 5 | | | 59 | 62 | 48 | 29.75 | 29.50 | 78 | 82 | SW | W | Cloudy | Rain | — |
| 6 | .10 | | 54 | 61 | 49 | 29.53 | 29.75 | 82 | 76 | WNW | NW | — | — | — |
| 7 | | | 56 | 63 | 49 | 29.83 | 29.94 | 75 | 81 | NNW | NE | Fair | Fair | Fine |
| 8 | | | 54 | 68 | 52 | 30.07 | 30.16 | 80 | 76 | NE | SE | — | Fine | — |
| 9 | | ○ | 63 | 70 | 54 | 30.18 | 30.18 | 75 | 77 | E | NNE | — | — | — |
| 10 | | | 60 | 71 | 51 | 30.18 | 30.11 | 77 | 73 | NE | NE | — | — | — |
| 11 | | | 60 | 73 | 53 | 30.06 | 30.02 | 71 | 79 | NE | NE | — | — | — |
| 12 | | | 59 | 73 | 53 | 30.01 | 30.03 | 76 | 82 | NNE | NE | — | — | — |
| 13 | | | 59 | 71 | 52 | 29.98 | 29.92 | 74 | 80 | ENE | ENE | — | — | — |
| 14 | | | 61 | 75 | 58 | 29.82 | 29.68 | 81 | 77 | NE | ENE | — | — | Sl. Rain |
| 15 | | | 59 | 69 | 60 | 29 64 | 29.63 | 92 | 88 | E | S | Cloudy | Cloudy | Cloudy |
| 16 | | | 62 | 67 | 59 | 29.61 | 29.64 | 90 | 89 | SSW | SE | — | — | — |
| 17 | | ☾ | 67 | 74 | 60 | 29.69 | 29.78 | 85 | 79 | W | SW | Fair | Fair | — |
| 18 | | | 63 | 73 | 57 | 29.85 | 29.85 | 87 | 87 | W | SW | — | Cloudy | — |
| 19 | | | 63 | 71 | 56 | 29.82 | 29.71 | 76 | 76 | W | SW | — | — | — |

The quantity of ain fallen in the month of May, was 2 inches and 12.100ths.

